# Acceptability of self-sampling for etiological diagnosis of mucosal sexually transmitted infections (STIs) among transgender women in a longitudinal cohort study in São Paulo, Brazil

**DOI:** 10.1016/j.bjid.2022.102356

**Published:** 2022-05-02

**Authors:** Daniel Jason McCartney, Thiago Félix Pinheiro, José Luis Gomez, Paula Galdino Cardin de Carvalho, Maria Amélia Veras, Philippe Mayaud

**Affiliations:** a London School of Hygiene & Tropical Medicine, Keppel Street, London WC1E 7HT, United Kingdom; b Faculdade de Ciências Médicas da Santa Casa de São Paulo, São Paulo, SP, Brazil

**Keywords:** Transgender women, Sexually transmitted infections (STIs), Self-sampling, Chlamydia trachomatis, Neisseria gonorrhoeae

## Abstract

This study conducted among transgender women in São Paulo, Brazil assessed the acceptability and suitability of screening sexually transmitted infections (STIs), such as *Chlamydia trachomatis* and *Neisseria gonorrhoeae*, by sampling multiple anatomical sites (i.e. urethral, anorectal, oropharyngeal, and neovaginal), and utilizing self- or provider-collection methods. First, a convenience sample of 23 cohort participants were recruited during a scheduled study visit between October and November 2018. Data collection was through a short investigator-led quantitative survey in Portuguese, and included presentation of investigator-designed, gender-neutral instructional diagrams to guide self-sampling. Three supplemental focus group discussions (FGDs) with a total of 30 participants guided by semi-structured script were conducted in Portuguese between September and October 2019. All participants reported being assigned male sex at birth and self-identified with a feminine gender identity at time of study. All survey respondents (100%; n = 23) indicated willingness to provide samples for STI screening during a future study visit. Preference was for self-collection of urine samples (83%; n = 19), urethral swabs (82%; n = 18), and anorectal swabs (77%; n = 17). A lower preference for self-collection of oropharyngeal swabs (48%; n = 11) was observed. Most respondents (78%; n = 18) indicated that they would not prefer specimens to be collected by a health professional, mainly due to ‘more privacy’ (72%; n = 13). All respondents indicated that they would feel comfortable to provide a self-collected sample based on instructional diagrams shown. In FGDs, although the collection by a health professional was described as a technically safer option for some participants, there was a preference for self-collection to avoid discomfort and embarrassment in exposing the body. Overall, this sub-study suggested acceptability among transgender women of introducing self-sampling for etiological diagnosis of STIs from potential infection sites. Uptake and usability will be explored further in a cross-sectional STI prevalence study of transgender women in Brazil.

## Introduction

Transgender women are known to be at high risk of HIV and other sexually transmitted infections (STIs). While HIV prevalence among transgender women is relatively well-studied, very little is known about other STIs, in particular bacterial STIs such as chlamydia and gonorrhoea.^1^^,^^2^ A recent systematic review found a limited number of studies that included data on syphilis, gonorrhoea, and chlamydia among transgender women, and estimated prevalence ranging from 1.4 to 50.4%, 2.1 to 19.1%, and 2.7 to 24.7%, respectively.^2^ Despite high prevalence of STIs in populations of transgender people, there remains limited clinical guidance tailored to STI screening, with most national protocols for STIs not providing any specific considerations.^2^^,^^3^

While syndromic management of STIs refers to the diagnosis and treatment based on common STI syndromes, etiological diagnosis of STIs provides a more definitive diagnosis by testing a sample of blood, urine, or swab-based specimen collection at relevant anatomical sites. This allows for better targeted treatment and improves antibiotic stewardship. For cis-gender women, sampling commonly includes urine collection and specimens collected by a health professional at endocervical and vaginal sites, while sampling urethral site by health professional or urine collection is common for cis-gender men. However, STI screening is often not routinely conducted at anorectal or oropharyngeal sites, leaving the possibility of undiagnosed infections, especially among certain populations with high prevalence of STIs. There is little evidence to guide routine screening in asymptomatic transgender women who have undergone vaginoplasty, and the role of vaginal specimens is currently unknown.^4^

In practice, transgender people may avoid screening procedures and physical examinations due to fear of discrimination, encountering health professionals who are inadequately trained, or personal discomfort with the visit or exam, and may prefer to collect their own specimens to allow for greater control over the screening process.^4^

Self-sampling, including urine collection and self-collected swabs (SCS), allows routine specimen collection without the need for a physical examination or provider-collected swabs (PCS). This provides a benefit both for efficiency of health professionals with limited time and capacity, as well as enabling those who may not access service due to actual or perceived requirement of a clinician needing to complete a physical examination.

Many studies have demonstrated that SCS have equivalent sensitivity and specificity to PCS for nucleic acid amplification testing (NAAT) for *Chlamydia trachomatis* and *Neisseria gonorrhoeae*,^5^^,^^6^ and self-sampling has become an important tool for expanding STI testing. With the potential to address common barriers including inaccessibility, inconvenience, embarrassment, and discomfort,^7^^,^^8^ self-sampling for STI diagnosis has been found to be a highly acceptable method among patients.^9^ A recent review found self-collection of samples increased uptake of STI testing services when compared to samples collected by a health professional.^10^

Past studies evaluating the validity, feasibility, and acceptability of SCS have focused primarily on vaginal swabs among cis-gender females and rectal swabs for MSM. Among MSM who collected their own rectal and pharyngeal samples, detection rates were found to be of equal or better accuracy than those of health professionals.^11^

For transgender people, there is insufficient evidence specific to SCS, with only one study identified from Boston, USA comparing the performance and acceptability of SCS and PCS for detection of high-risk genotypes of the human papillomavirus (HPV) among transgender men (self-identified as men, assigned female at birth).^12^ No relevant studies with transgender women were found at the time of study.

Visual aids are commonly used to help support SCS, including instructional diagrams or videos, often designed for cis-gender males and females.^9^^,^^13^, ^14^, ^15^ These illustrative tools can be modified for different settings and co-developed with target populations for increased understanding and acceptability.^16^ However, no published examples were found of self-guided diagrams with gender inclusive or gender-neutral instructions to enable self-collection of anatomically diverse populations.

The objective of this study was to assess the acceptability and suitability of screening STIs, such as *C. trachomatis* and *N. gonorrhoeae*, among transgender women by sampling multiple anatomical sites (i.e. urethral, anorectal, oropharyngeal and neovaginal), and utilizing SCS or PCS.

## Methods

### Study population

The study was conducted among transgender women participating in a longitudinal cohort study aiming to determine HIV, syphilis, and viral hepatitis seroprevalence in São Paulo, Brazil. Briefly, the TransNational Study aimed to enroll 550 transgender women aged 18 years and over in the metropolitan area of the city of São Paulo following a respondent-driven sampling (RDS) methodology.^17^ The opportunity was afforded to interview a sample of volunteers to determine the acceptability and practicability of mucosal STI screening in addition to blood samples collected for serological testing.

This sub-study included mixed quantitative and qualitative methodologies through convenience sampling. Consecutive potential participants from the existing cohort study were invited during a scheduled study visit over a two-week period with a target enrolment of 20 participants to complete a quantitative survey. Following the initial results from the survey, additional focus group discussions were arranged with cohort participants. Written informed consent was obtained from all participants.

### Quantitative survey

Data was collected using a short investigator-led questionnaire in Portuguese. This included the presentation of investigator-designed, gender-neutral instructional diagrams for self-sampling utilizing oropharyngeal, anorectal, and vaginal swabs, and provision of urine samples. Participants received information about the proposed addition of STI screening to the cohort study, and the investigator received informed consent to conduct the additional survey. No samples were provided in this sub-study.

### Focus group discussions

Focus group discussions (FGDs) were conducted in Portuguese and guided by a semi-structured script to discuss the acceptability of self-sampling versus collection by a health professional of oropharyngeal, urethral, and anorectal samples for the diagnosis of STIs. Thematic analysis of transcripts was conducted in Portuguese, with key quotes translated to English by the investigators.

## Results

### Study participants

A total of 23 participants from the cohort study were invited to this sub-study between 29 October to 13 November 2018, during one of their scheduled study visits (ranging from first to fifth visit), and none declined. Participant characteristics are presented in Table 1. Participants’ ages ranged from 18 to 45 years, with a median age of 27 years. All reported residing in the city of São Paulo, except one participant residing elsewhere in the state of São Paulo. They reported being assigned male sex at birth and identified with a feminine gender identity at the time of study. Of the 23 participants, one confirmed having had genital or lower surgery (gender-affirmative surgery) to remove their male genitalia.

**Table 1:** Participant characteristics of the quantitative survey respondents (N = 23).

Characteristics	Summary statistics [*n* (%)]
Age (years)	
Mean (SD)	27.8 (7.6)
Median (range)	27 (18-45)
Gender identity	
Woman (*mulher*)	7 (30.4)
Transsexual woman	11 (47.8)
*Travesti*	4 (17.4)
Other female gender identity	1 (4.3)
Residence	
São Paulo city	22 (95.7)
Sorocaba	1 (4.3)
Study visit	
First	1 (4.3)
Second	3 (13.0)
Third	8 (34.8)
Fourth	5 (21.7)
Fifth	6 (26.1)
Gender-affirmation surgery	
Yes	1 (4.3)
No	22 (95.7)

Three FGDs with a total of 30 participants were conducted at the study clinic in São Paulo between 24 September and 1 October 2019. The first group was composed of transgender women who completed high school education; the second group was composed of transgender women sex workers without completed high school education; and the third group was composed of transgender women with different professional activities and different levels of education.

### Previous experience of STI sampling

Most survey respondents (70%; n = 16) stated that they had never had an STI test that required a urine sample or swab, while one respondent (4%) was uncertain. Over a quarter (26%; n = 6) indicated that they had tested in the past, with five (22%) indicating experience of oral swabs, two (9%) of urethral swabs, and one (4%) of rectal swabs, while one (4%) did not provide response of anatomical site. In total, three (13%) responded that these were self-collected, two (9%) collected by professional, and one (4%) reported collection both by self and professional.

### Sampling preference

All survey respondents were asked to indicate their preference of method for providing samples if they were to visit a clinic for STI testing. For all sample methods (excluding vaginal), an overall preference was for these samples to be self-collected (Table 2). In order of preference for self-collection, this was greatest for urine sample (83%; n = 19), urethral swab (82%; n = 18); and rectal swab (77%; n = 17). Only two sampling methods had preference for provider-collected: oral swab (13%; n = 3) and oral rinse (9%; n = 2). While some indicated no preference for each of the sampling methods, none expressed being uncertain.

**Table 2:** Preference for self-collected versus provider-collected samples for STI testing (N = 23).

Sample type	Self-collected	Provider-collected	No preference	Unsure	Total responses
Urethral swab	18 (82%)	0	4 (18%)	0	22
Oral swab	11 (48%)	3 (13%)	9 (39%)	0	23
Oral rinse	11 (50%)	2 (9%)	9 (41%)	0	22
Rectal swab	17 (77%)	0	5 (23%)	0	22
Vaginal swab	0	0	1 (100%)	0	1
Urine sample	19 (83%)	0	4 (17%)	0	23

FGD participants considered that urine collection and oral (oropharyngeal) swab collection were acceptable and straightforward procedures. Participants’ preferences for self-collected or provider-collected oral swabs diverged:

*“I'd rather I do it myself.”*

*“I prefer the professional, I feel safer.”*

*“If you're a professional, I'd rather not risk [self-collecting].”*

(Participants from FGD3)

*“That depends on who the professional is.”*

(Participant from FGD2)

The collection of specimen samples from the penile urethra or vagina generated much divergence in the focus groups, although no participant stated that they would refuse to do so. The preference of some participants for self-collection was related to their discomfort of exposing their naked body and having their genitalia handled by a medical professional:

*“I already find it very embarrassing the person [professional] is doing this down there on you.”*

*“I think it's a boring exam.”*

*“I think it would be a very intimate thing of the person, it would have to be you could do it yourself.”*

*“It gives shame.”*

(Participants from FGD2)

*“I, in my uniqueness... he [the professional] never saw my [genitals]. And then, I'll get there... I can't do it.”*

(Participant from FGD1)

The collection of anorectal samples also generated divergence, although it seemed less controversial than collection from genitals (penile urethra or vagina). In the third FGD, some participants stated that they would refuse to perform this collection:

*“I'd stop doing it because I wouldn't feel comfortable.”*

(Participant from FGD3)

However, the preference for self-collection was more expressive, although some participants stated that they would not have resistance to let the professional perform the collection:

*“I think it's unnecessary for a professional to do this kind of action. Why couldn't you do it yourself, walk into a small room and do it?”*

(Participant from FGD2)

*“Everything is an option. If [the professional] gives me the option to go there and collect, fine. If I don't have [that] option, I will let [the professional] collect.”*

(Participant from FGD1)

### Acceptability of sampling

All survey respondents were asked if they would be willing to provide samples for screening of other STIs during a future study visit. All provided a positive response (100%; n = 23) and indicated that they would feel comfortable collecting samples by themselves if received information on how to collect (n = 21; 2 missing).

When asked if they would prefer samples to be collected by a health professional, two (9%) indicated that they would prefer, while three (13%) indicated no preference. For the two respondents who indicated that they would prefer samples to be collected by a health professional, they explained that this was due to ‘preference by trained professionals’, and ‘afraid to take the wrong exam’.

**Fig. 1:**
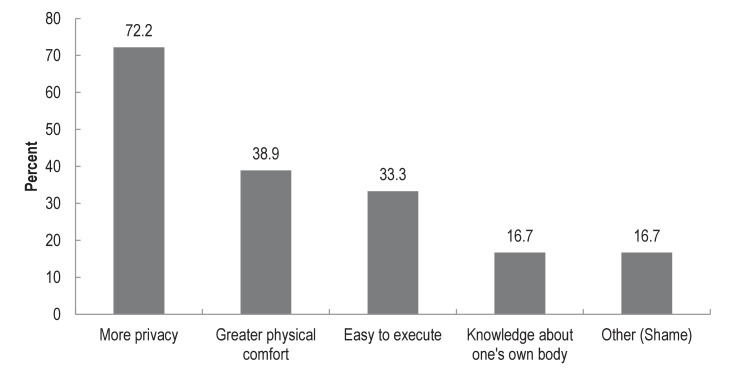
Reasons for preferring self-collected samples for STI testing (N = 18).

Most respondents (78%; n = 18) indicated that they would not prefer specimens to be collected by a health professional. The reasons for preferring self-collection are illustrated in Figure 1. The main reason provided was for ‘more privacy’ (72%; n = 13). Other reasons were for ‘greater physical comfort’ (39%; n = 7), ‘easy to execute’ (33%; n = 6); and ‘knowledge about one's own body’ (17%; n = 3). Of respondents who indicated ‘other’ reason (17%; n = 3), all explained due to ‘shame’.

During the FGDs, one participant told of an experience in which she was satisfied with the possibility of self-collection offered:

*“I went to take an exam, and he [the professional] said: If you see that you will be embarrassed to show your organ to me, I close the curtain and give you a cotton swab for you to do. Then I said, I prefer it that way. Did you understand?? He was a gentleman, but I wasn't going to make it because he also had a girl in his office who stays with him. Then he pulled the curtain, then I took the cotton swab.”*

(Participant from FGD2)

The participants who showed preference for the collection by a health professional stated that they felt more confident because it was perceived that the professional would have more technical knowledge and a greater ability to perform the procedure correctly:

*“I prefer with the professional, I feel safer.”*

(Participant from FGD3)

*“It's just that sometimes you can do it one way and the doctor does it another.”*

(Participant from FGD2)

*“He's the doctor, he'll know where to go.”*

(Participant from FGD1)

Faced with the argument that the professional has more technical knowledge to perform the collection, one participant proposes that guidance material for self-collection be offered:

*“Just show a little video like that running [the swab] in the little head [of the penis].”*

(Participant from FGD1)

Although the gender of the health professional did not seem to be a relevant issue for some participants, others indicated that they would have different reactions to men or women:

*“I feel uncomfortable whether [the health professional] is a woman or a man.”*

(Participant from FGD1)

*“I think I would let [the health professional collect sample] if it was a professional woman, but if it was a man, I'd be ashamed.”*

*“I prefer a woman, because it's better than a professional man, you know? It's because there are some doctors who even have a prejudice, you understand? And also he won't say 'I'm prejudiced or not' [because] he's a professional. (...) You have to take the exam, you have to feel good. So I prefer a professional woman.”*

*“I like being served by a man, my private parts are man's, not a woman's.”*

(Participants from FGD2)

One participant suggested that the ideal would be for a transgender professional to perform the collection and received great agreement from the group.

In the second FGD composed of transgender women sex workers, there was a discussion about the different relationships between the exposure of the body in the context of sex work and in the context of health care:

*“It's because, for people who lose their aesthetics, who feel ashamed, being naked in front of a [client] is different from being naked in front of a professional. (...) [For] people who work at night it is different to be naked with a [client] and be at ease than the professional who is a doctor.”*

*“I lost this fear [of showing my naked body] through the customers, because (...) when I go into the room, I have to take off my clothes with the guy I don't even know, you know? I think I'd easily let [the health professional] use the swab, because I think it breaks all the taboos, we work with the body at night, and we don't have to be ashamed to expose ourselves to the client. So why are you going to be ashamed to exposure yourself to a man who is going to give you the cure for what you're looking for?”*

(Participants from FGD2)

### Self-sampling instructional diagrams

A total of 20 survey respondents were shown the instructional diagrams for self-sampling and asked to indicate their level of understanding. Overall, the majority of respondents provided positive responses of understanding (Table 3).

**Table 3:** Stated level of understanding of instructional diagrams for self-sampling (N = 20).

Sample type	Very easy	Easy	Difficult	Very difficult	Total responses
Oral swab	9 (45%)	11 (55%)	0	0	20
Rectal swab	8 (40%)	10 (50%)	2 (10%)	0	20
Vaginal swab	1 (100%)	0	0	0	1
Urine sample	10 (50%)	9 (45%)	1 (5%)	0	20

All respondents indicated that the oral swab was *easy* (55%; n = 11) or *very easy* (45%; n = 9) to understand. For the urine sample, most respondents indicated that it was *very easy* (50%; n = 10) or *easy* (45%; n = 9) to understand, while one (5%) indicated that it was *difficult* to understand. For the rectal swab, most respondents indicated that it was *easy* (50%; n = 10) or *very easy* (40%; n = 8) to understand, while two (10%) indicated that it was *difficult* to understand. Only one participant was eligible to review the vaginal swab diagram and indicated that it was *very easy* to understand.

All participants were asked whether, based on the instructional diagrams shown, they would feel comfortable to self-collect the sample. All provided an affirmative response, with one explaining that they would feel less comfortable with the rectal samples as they felt ‘[it] would be difficult to collect’.

## Discussion

With accuracy and acceptability of self-collected samples for STI testing demonstrated more generally in other studies,^5^, ^6^, ^7^, ^8^ this study provided much needed additional evidence of acceptability and suitability of self-sampling specifically among transgender women, and from different potential infection sites.

As stigma and discrimination may pose additional barriers to the utilization of health services among transgender people,^18^ this study provided an indication that self-collection of samples may help to alleviate some discomfort when encountering health professionals. Further research is needed to better understand the reasons for avoidance of testing among transgender women, and to understand whether self-collection helps increase the utilization of STI testing.

However, there was limited evidence to suggest a perception that specimen collection by a health professional was the norm for cis-gender women and was therefore the sampling method some participants stated they would prefer. This could be a powerful part of gender affirmation, whereby transgender women are not wanting to be treated differently from cis-gender women.

Visual aids are important to guide effective self-collection, with imagery co-created with the target population critical to ensure suitability and acceptability.^16^ The novel gender-neutral instructional diagrams that were piloted in this study received positive responses of understanding to enable self-collection of samples, with further development and testing warranted.

Overall, transgender people remain an understudied population with a paucity of evidence-based interventions tailored to their unique needs. There remains an expressed lack of screening and other clinical guidance specifically tailored to transgender populations, with more research needed to inform appropriate and effective strategies/interventions to reduce risk of STI acquisition and transmission.^2^^,^^3^

One limitation of this study was that it did not include actual sample collection. With more data needed on acceptability of self-sampling in real-life settings, uptake and usability will be explored further in a large cross-sectional STI prevalence study of transgender women in Brazil (TransOdara). The research findings will have important policy and public health implications in Brazil and internationally by informing specific STI-related recommendations for transgender women including etiological screening and management of urethral, anorectal, oropharyngeal and neovaginal infections.

## Ethical aspects

The TransNational Study protocol was reviewed and approved by the Brazilian National Commission on Research Ethics (CONEP-CNS, #1880217), the Ethical Review Committee of the Centro de Referência e Treinamento em DST/AIDS (CRT DST/AIDS), and the Internal Review Board of the University of California San Francisco. The protocol for this sub-study was reviewed and approved by the London School of Hygiene & Tropical Medicine (LSHTM) Ethics Committee. Informed consent was obtained from all individual participants included in the study. All procedures were carried out in accordance with the Declaration of Helsinki.

## Conflicts of interest

All authors declare no conflicts of interest.
